# Workplace Health Interventions Targeting Cardiovascular Diseases and Diabetes Mellitus for Blue-Collar Workers: Protocol for a Scoping Review

**DOI:** 10.2196/74248

**Published:** 2025-10-17

**Authors:** Chiara Di Girolamo, Agnieszka Lipiak, Matteo Franco, Krzysztof Kaczmarek, Guillermo Barreres-Martín, Piotr Romaniuk, Carles Muñoz-Alfonso, Andrea Arroyo-Álvarez, Federica Turatto, Daznia Bompart Berroterán, Ewelina Chawłowska

**Affiliations:** 1 Department of Clinical and Biological Sciences Centre for Biostatistics, Epidemiology, and Public Health University of Turin Orbassano, Turin Italy; 2 Department of Preventive Medicine Laboratory of International Health Poznan University of Medical Sciences Poznan Poland; 3 Department of Health Policy Faculty of Public Health Medical University of Silesia in Katowice Bytom Poland; 4 INCLIVA Biomedical Research Institute Valencia Spain

**Keywords:** blue-collar workers, cardiovascular disease, diabetes mellitus, workplace health promotion, workplace health interventions, chronic disease prevention, occupational health, public health

## Abstract

**Background:**

Cardiometabolic diseases, such as cardiovascular diseases (CVD) and diabetes mellitus (DM), represent a global health issue, causing significant premature mortality and disability. Blue-collar workers, who often face a higher exposure to risk factors (eg, smoking, poor diet, and lack of physical activity), are particularly vulnerable to these diseases. Workplace health promotion plays a crucial role in mitigating this risk, yet the scope and the characteristics of interventions targeting this specific workforce remain unclear.

**Objective:**

The objective of this scoping review will be to assess the extent and characteristics of workplace health promotion interventions aimed at preventing cardiometabolic diseases and their risk factors in blue-collar workers.

**Methods:**

The review will follow the JBI methodology for scoping reviews. A search of MEDLINE, Scopus, Embase, and the Cochrane Database as well as grey literature will be conducted. The search strategy is designed to capture relevant studies published since 2014 in English, Spanish, Italian, and Polish. Eligible study designs include experimental design (eg, randomized controlled trials), observational studies (eg, longitudinal studies), qualitative research, and mixed-method approaches as well as other formats present in grey literature (eg, reports). This review will include studies focusing on health promotion interventions involving adult blue-collar workers (aged ≥18 years) currently employed, with at least 60% of participants being blue-collar employees. The interventions of interest are workplace health promotion strategies targeting CVD, DM, or their risk factors, such as hypertension, obesity, and smoking. Each paper retrieved will be screened for inclusion by at least 2 reviewers. Disagreements between the reviewers will be resolved through discussion with the other reviewers. Studies focusing on noninterventional contexts, mental health, or ergonomic safety will be excluded. Data will be extracted and analyzed using qualitative and quantitative methods, with a focus on intervention types, participant characteristics, and outcomes.

**Results:**

The study began in March 2024 and the full scoping review report is expected to be ready in September 2025.

**Conclusions:**

The scoping review presented in this protocol can contribute to filling the knowledge gap by mapping the current state of workplace health promotion interventions aimed at preventing CVD and DM and modifying relevant risk factors in the blue-collar workers’ group.

**Trial Registration:**

Open Science Framework hdrq4; https://osf.io/hdrq4

**International Registered Report Identifier (IRRID):**

DERR1-10.2196/74248

## Introduction

Cardiometabolic diseases, including cardiovascular diseases (CVD), diabetes (DM) and nonalcoholic fatty liver disease [1], are a leading health problem on a global scale, being the most common cause of premature mortality and disability [2,3]. In 2019, it was estimated that over half a billion people in the world suffered from CVD, and 18.2 million died from this cause [4]. In the case of DM, the number of patients was estimated at 529 million people; over the years 2010-2021, DM experienced the most rapid growth among the causes of health loss after adjusting for age and population size [3]. Although older age is one of the most important risk factors for cardiometabolic diseases [5,6], it is estimated that up to one-third of deaths due to circulatory system diseases occur before the age of 70 [7]. Moreover, available research indicates that CVD and DM are some of the main causes of early exit from paid work [8,9], making it a burden to the health system as well as to the national, regional, and global economy.

To address this challenge, several initiatives have been launched to allow for a better understanding of the determinants leading to CVD and DM and increase the effectiveness of preventive measures both on the individual and social levels. One such initiative is the Joint Action on Cardiovascular Diseases and Diabetes (JACARDI), implemented under the EU4Health Program of the European Commission [10]. JACARDI’s objective is to support EU countries in reducing the burden of CVD and DM and related risk factors while ensuring health systems’ sustainability and equity. JACARDI aims at enhancing and promoting the implementation of best practices and pilot testing of innovative practices throughout the whole patient journey. One of the JACARDI Work Packages aims at supporting the labor participation of people living with noncommunicable diseases, particularly with CVD and DM and related risk factors, by identifying issues affecting labor participation and testing systemic and organizational solutions to improve inclusion, retention, and return to work, and support well-being at the workplace. Within the broader JACARDI objectives, this scoping review will focus specifically on CVD and DM prevention.

Considering how heterogenous working population is in terms of exposure to risk factors for CVD and DM, this scoping review will focus on blue-collar workers, a group of employees that generally includes people who perform low-complexity manual work, usually not requiring advanced qualifications or higher education [11]. Numerous studies indicate that these workers are more likely to be diagnosed with obesity, hypertension, and dyslipidemia [12,13] and are more exposed to behavioral risk factors associated with lifestyle, such as smoking, alcohol consumption, poor eating habits, and lower levels of physical activity [14,15]. For this reason, health promotion is pivotal in preventing CVD and DM and mitigating their effects. Indeed, in the case of the working-age population, workplace health promotion, understood as combined efforts of employers, employees, and society to improve the health and well-being of people at work [16], becomes a very important vehicle for change. Therefore, identifying the scope and characteristics of interventions undertaken in the workplace environment to tackle the burden of CVD and DM risk factors is a necessary first step in the way to fully exploiting the potential of the working environment to improve the population’s health.

The scope of literature on health promotion interventions aimed at preventing CVD and DM in blue-collar workers is currently unknown. A preliminary search of PubMed, Scopus, Embase, and the Cochrane Database of Systematic Reviews was conducted and no current or underway systematic reviews or scoping reviews on this specific topic were identified. During the search, several cases of reviews with similar topics were found that differ from this scoping review due to their reference to the general population of employees [17-21] or their focus on one type of intervention (ie, behavioral interventions) or disregarding “diabetes” as a keyword [17,21,22].

Providing a critical review of specific workplace health interventions targeting blue-collar workers can help stakeholders identify which actions can be rolled out to prevent CVD and DM in workplace settings. For these reasons, the objective of this scoping review is to assess the extent and characteristics of workplace health promotion interventions aimed at preventing CVD, DM, and their risk factors in blue-collar workers, consistent with JACARDI’s objectives. The ultimate aim is to provide a comprehensive overview of the most frequently implemented interventions and prevention strategies in addressing the risk factors for CVD and DM and to examine potential variations in intervention approaches across different geographical settings and sectors.

## Methods

### Overview

This scoping review will be conducted in accordance with the JBI methodology for scoping reviews [23] and will be reported following the PRISMA-ScR (Preferred Reporting Items for Systematic Reviews and Meta-Analyses extension for Scoping Reviews) checklist [24]. The identification and the examination of the research question is based on the Participant, Concept, Context (PCC) framework. This scoping review was registered on Open Science Framework. The planned study period is May 2024 to September 2025.

### Participants

This review will include studies that involve adult (aged ≥18 years) blue-collar workers (with jobs listed by the International Labor Organization under codes 6, 7, 8, and 9) who are currently active in the labor setting. In case of mixed study groups including blue-collar, white-collar, and pink-collar workers, we will include a study when blue-collar workers constitute at least 60% of participants. Studies where the proportion of blue-collar workers is impossible to determine will also be excluded. Studies involving retired or sick workers or unemployed persons will be excluded.

### Concept

This scoping review will focus on health promotion interventions that target CVD and DM disease or their risk factors (ie, hypertension, dysglycemia, dyslipidemia, overweight and obesity, smoking, alcohol, physical inactivity, and unhealthy diet including insufficient consumption of fruits and vegetables) [7,25]. We are interested in studies focusing on the evaluation of the results or the methodology of the following health promotion interventions: (1) health education, (2) prevention of risk factors and diseases or elimination of exposures (primary prevention), (3) disease or risk factor detection or screening (secondary prevention), and (4) health policy changes (eg, changes in infrastructure). We will consider health promotion interventions that entail both voluntary and compulsory participation.

We will exclude studies that report the context of an intervention (without direct connection to intervention results or the methodology), and those that report interventions primarily focusing on: (1) mental health (eg, stress, burnout, and depression), (2) musculoskeletal problems, (3) sun or skin protection, (4) violence or harassment prevention, (5) infections (vaccination and hygienic behaviors), (6) sexual behaviors, and (7) pollution.

### Context

This review will consider studies that involve health promotion interventions carried out or initiated in workplaces among blue-collar workers. The review will not be limited to any specific country or work setting.

### Types of Evidence

This scoping review will consider empirical studies describing interventions that present quantitative, qualitative, or mixed-method results. Both experimental and quasi-experimental study designs including randomized controlled trials, nonrandomized controlled trials, before and after studies, and interrupted time-series studies will be eligible. Qualitative studies will be considered including, but not limited to, designs such as ethnography and action research, provided they involve an intervention with measurable results.

Studies published in English, Spanish, Italian, or Polish will be included, as these are the languages the participating researchers are proficient in. Additionally, to cover the last 10 years of literature and limit variations in technology availability, only studies published since 2014 will be considered.

A summary of the eligibility criteria is reported in Table 1.

**Table 1:** Eligibility criteria.

Category		Criteria
**Participants**		
	Include	Blue-collar workers (ILO^a^ codes 6, 7, 8, and 9). Adults (aged ≥18 years). Current active workers. In mixed study groups including blue-collar, white-collar, and pink-collar workers, only those where blue-collar workers constitute at least 60% of participants.
	Exclude	Retired workers. Workers on sick leave. Unemployed individuals.
**Concept**		
	Include	A past or ongoing health promotion intervention targeting CVD^b^ and DM^c^ or their risk factors (ie, hypertension, dysglycemia, dyslipidemia, overweight and obesity, smoking, alcohol, physical inactivity, and unhealthy diet). Interventions including health education, prevention of risk factors and disease, elimination of exposures, screening, and health policy changes.
	Exclude	Studies focused on the context of an intervention (not directly connected to intervention results or methodology). Interventions primarily focusing on mental health (stress, burnout, and depression), musculoskeletal problems, sun or skin protection, violence or harassment prevention, infections (vaccination and hygienic behaviors), sexual behaviors, and pollution.
**Context**		
	Include	Workplace health interventions (taking place at the workplace or initiated at the workplace but performed outside).
	Exclude	—^d^
**Study characteristics**		
	Include	Empirical studies (quantitative, qualitative, and mixed-method studies). Original research. Articles, books, reports, and grey literature. Published online and free to access. Written in English, Italian, Polish or Spanish. Published after 2013.
	Exclude	Study protocols. Reviews, systematic reviews, scoping reviews, and umbrella reviews.

^a^ILO: International Labor Organization.

^b^CVD: cardiovascular disease.

^c^DM: diabetes mellitus.

^d^No specific exclusions.

### Search Strategy

The search strategy aims at locating published documents. A 3-step search strategy will be used in this review. First, an initial limited search of MEDLINE (PubMed) and Scopus will be undertaken to identify articles on the topic. To ensure a comprehensive search, a variety of terms associated with blue-collar occupations will be incorporated. Additionally, the International Classification of Occupations will be used to delineate jobs classified as blue-collar. Specifically, categories 6, 7, 8, and 9 will be selected based on previous reviews [26] that identified these categories as blue-collar. In addition, diverse terms describing interventions and each specific outcome of interest will be included. The search strategy will be developed through an iterative process involving the creation and testing of multiple search strings including terms with various degrees of generality. The researchers will prepare an initial list of general terms reflecting the elements of the PCC framework (eg workplace, intervention, blue-collar, and cardiovascular). Next, they will begin the search in the 2 databases using combinations of the initial terms. If any relevant publications are identified this way, their abstracts and keywords will be screened for additional terms (eg, synonyms, alternative spellings, and subgroups of the initial concepts). All terms identified in the process will be added to the relevant PCC categories and pretested in strings. The final search strings will be selected from among those yielding a manageable number of results with a big proportion of potentially relevant results. This means that strings that will retrieve approximately 200–300 records and more than 60% entries will be potentially relevant upon title and abstract screening and kept. This threshold is intended to ensure feasibility while preserving comprehensiveness. Finally, the full search strategy will be adapted for each of the included databases: MEDLINE (PubMed), Scopus, Embase, and Cochrane Library (Cochrane CENTRAL). The final search string will be formulated to ensure that the included terms are located specifically within the title or abstract fields. As an example, the search keywords applied to the Embase Database on June 6, 2024, are reported in Textbox 1.

To ensure a comprehensive review of relevant sources not indexed in traditional databases, we will incorporate sources of grey literature, encompassing international organizations such as the World Health Organization, the European Agency for Safety and Health at Work, and the National Institute for Health and Care Excellence. We will also look for relevant national resources from Poland, Italy, and Spain, which are the countries of the researchers carrying out this scoping review and involved in JACARDI. These include governmental and institutional databases on health, labor, and occupational safety, such as the Polish National Institute of Occupational Health, the Italian Ministry of Labor, and the Spanish National Institute of Health and Safety at Work. If any relevant search terms are identified in these sources, they will be added to the search strings pretested in the 4 academic databases.

**Search keywords applied to the Embase Database on June 6, 2024.**

1. manual OR unqualified OR unskilled OR semi-skilled OR middle-skilled OR low-skilled OR low-wage OR “low wage” OR forestry OR fishery OR agricultural OR hunting OR craft OR building OR metal OR handicraft OR printing OR equipment OR electronics OR telecommunications OR “food processing” OR woodworking OR garment OR ship OR laundry OR cleaning OR mining OR construction OR manufacturing OR transport OR storage OR “food preparation” OR “street sales” OR elementary. ti,ab.
2. workers OR employees OR workforce OR staff OR personnel OR labo*rers. ti,ab.
3. 1 AND 2.
4. blue-collar OR bluecollar OR “blue collar” OR labo*rers OR wage-earn* OR gardeners OR “crop grow*” OR “animal produc*” OR “crop produc*” OR hunters OR trappers OR farmers OR fishers OR gatherers OR finishers OR painters OR cleaners OR moulders OR welders OR blacksmiths OR toolmakers OR mechanics OR repairers OR printers OR installers OR electricians OR “wood treaters” OR “cabinet-makers” OR “plant operator*” OR “machine operators” OR assemblers OR drivers OR “deck crew*” OR helpers OR “street vendors” OR “refuse workers”. ti,ab.
5. 3 OR 4.
6. workplace*, work-place OR worksite*, work-site OR “work place” OR “work site” OR occupational. ti,ab.
7. “intervention” OR “program” OR “programme” OR “project” OR “trial” OR randomi?ed OR “RCT” OR “experiment”. ti,ab.
8. health OR wellness OR well-being OR wellbeing. ti,ab.
9. 6 AND 7 AND 8.
10. whp. ti,ab.
11. 9 OR 10.
12. 5 AND 11.
13. limit 12 to original research.
14. Limit 13 to publication year from 2014 to 2024.

### Study Selection

All identified citations will be collated and uploaded into Covidence systematic review software (Veritas Health Innovation), and duplicates will be removed. Then, each title and abstract will be screened by 2 independent reviewers following the inclusion criteria for the review. Potentially relevant resources will be sent to the full text analysis section, where they will be thoroughly evaluated against the inclusion criteria by 2 independent reviewers. Reasons for exclusion of sources at the full text analysis will be recorded and reported in the scoping review. Any disagreements between reviewers at each stage of the selection process will be resolved by involving a third reviewer.

**Figure 1:**
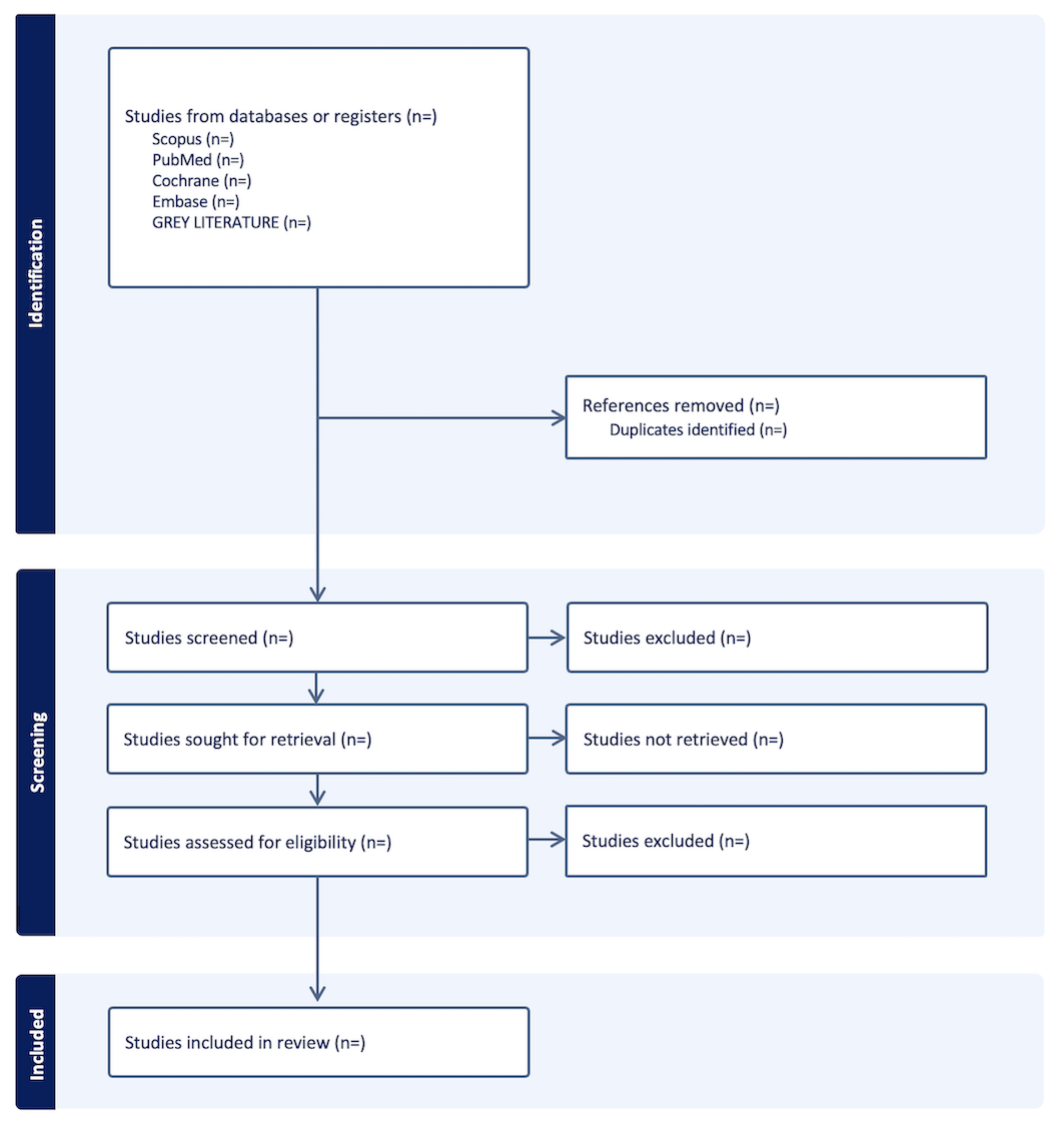
Preliminary PRISMA (Preferred Reporting Items for Systematic Reviews and Meta-Analyses) flow diagram.

A preliminary version of the PRISMA flow diagram [27] is shown in Figure 1 (checklist provided in Multimedia Appendix 1). The full version including total results of the study inclusion process will be reported in the final scoping review.

### Data Extraction

Data will be extracted from papers included in the scoping review by 2 independent reviewers using a data extraction table developed in Covidence by the authors; a third reviewer will check for consistency and will resolve any disagreements. The extracted data will encompass specific information regarding the participants, concept, context, study methods, source type, type of intervention, target group, and key findings pertinent to the review question. To ensure the proper functioning and suitability of the data extraction table, an initial extraction will be conducted on 5 of the evidence sources selected and then the appropriateness of the extraction table will be discussed. The following data will be extracted:

- Authors and date
- Title
- Geographical location
- Source type
- Intervention title
- Language
- Aim
- Type of research
- Study design
- Industry
- Professions
- Targeted population
- Number of participants
- Number of blue-collar workers
- Mean age and percentage of men
- Type of intervention
- Theoretical framework
- Diseases targeted
- Risk factors targeted
- Intervention content
- Findings

The data extraction table will be modified and revised as necessary throughout the process of extracting data from each included evidence source. Any modifications will be detailed in the scoping review.

### Data Analysis and Presentation

We will use quantitative and qualitative content analysis to document the intervention characteristics, participant characteristics, and reported outcomes. The unit of the analysis will be the intervention. We will present the extracted data graphically along with a narrative summary, which will accompany the results and will describe how the results relate to the review’s questions and objectives. For intervention characteristics, we will focus on type of intervention, implementation year, intervention setting, and diseases and risk factors targeted. For participant characteristics, we will focus on gender, age, and profession, whereas for the reported outcomes specifically, we will focus on the health-related changes (especially improvements) identified in blue-collar workers.

### Ethical Considerations

No ethical approval is required for this scoping review since it involves the analysis of already published data. The results of this study will be published in a peer-reviewed journal and discussed at scientific conferences. A summary of the study findings will also be developed to inform the work of the pilots of the work package on labor participation of people living with noncommunicable diseases within JACARDI.

## Results

The study began in March 2024 and the full scoping review report is expected to be ready in September 2025.

## Discussion

This protocol outlines the methods for a scoping review aimed at mapping and thoroughly describing health promotion interventions implemented or initiated in the workplace. While the review will share its focus on blue-collar workers with a few previous studies [22,26,28,29], it will differ by embracing all kinds of workplace interventions and by limiting its scope to those targeting CVD, DM, and their risk factors. The review will provide an insight into which risk factors and diseases are more frequently targeted, as well as which strategies are more frequently implemented. Moreover, it will highlight potential heterogeneity in interventions carried out in different geographical locations or in different sectors. This will be useful to guide pilots within JACARDI and to provide policymakers and companies with a better understanding of potential interventions for CVD and DM prevention.

The scoping review has some potential limitations. First, the review will be restricted to publications in English, Italian, Spanish and Polish, reflecting the languages spoken by the research team. This might result in underrepresentation of studies from other countries, notably from studies carried out in Asia, Africa, or South America. Moreover, by excluding interventions targeting mental health and musculoskeletal disorders, we may have missed integrated health promotion programs common in blue-collar workplaces. A quality assessment of the studies included is not planned as part of the scoping review. Although it might provide interesting information, it is beyond the scope of this study, which aims to describe the scope and content of the interventions described in the studies.

Despite the increasing implementation of workplace health promotion programs, there is currently a lack of synthesized evidence on their scope, characteristics, and effectiveness for this specific workforce. Given the significant burden of CVD and DM on public health and the global economy, it is crucial to better understand how occupational interventions can reduce these risks in vulnerable working populations. The scoping review can contribute to filling the knowledge gap by mapping the current state of workplace health promotion interventions aimed at preventing CVD and DM and modifying relevant risk factors in the blue-collar worker group.

## Appendix

Multimedia Appendix 1 Preliminary PRISMA-ScR checklist.

## Abbreviations

CVD: cardiovascular diseases
DM: diabetes mellitus
JBI: Joanna Briggs Institute
PCC: Participant, Concept, Context framework
PRISMA: Preferred Reporting Items for Systematic Reviews and Meta-Analyses
